# Eliminating Trachoma by 2020: Assessing Progress in Nigeria

**DOI:** 10.7759/cureus.9450

**Published:** 2020-07-29

**Authors:** Mustafa A Nasir, Fayez Elsawy, Abdulaziz Omar, Shah O Haque, Rans Nadir

**Affiliations:** 1 Medicine, Manchester University, Manchester, GBR; 2 Medicine, Imperial College London, London, GBR

**Keywords:** trachoma, get2020, nigeria, safe

## Abstract

Trachoma is a neglected tropical disease that causes an eye infection which can lead to blindness if left untreated. In 1998, the World Health Organisation (WHO) launched a new goal to eradicate trachoma by 2020. Over the years, in partnership with the WHO, an effective strategy plan was devised to help tackle and control the disease. This involved surgery for trichiasis, antibiotic treatment, facial cleanliness, and environmental improvement (SAFE). Consequently, the number of people affected by trachoma has significantly decreased in recent times. Despite this, trachoma remains a major public health concern in 44 countries worldwide, including Nigeria. Although improvements have been seen throughout Nigeria, the disjointed application of the SAFE strategy has delayed progress compared to other countries. Providing quality treatment to those with trachoma, in addition to improving preventative measures are challenges faced throughout the country. However, a multi-pronged approach emulating the methods of other countries is recommended to achieve trachoma elimination. This review aims to evaluate the progress and challenges faced in Nigeria with regards to eliminating trachoma.

## Introduction and background

Trachoma is one of the major causes of preventable loss of vision globally with an estimated 1.9 million people blinded or visually impaired [1]. Ocular infection with Chlamydia trachomatis initially causes inflammation of the conjunctiva. Repeated exposure to the bacterium without adequate treatment causes the upper eyelid to turn inwards which is known as trachomatous trichiasis. Ultimately, this results in scarring of the eye leading to the irreversible eye pathology associated with trachoma [2]. The main form of disease transmission occurs by direct contact between individuals who spread infective droplets from the eyes and nose, but the disease can also spread by fomites and flies. For this reason, trachoma tends to manifest among young children who lack facial hygiene and mothers who have high contact hours with their offspring [3].

Trachoma falls within the category of neglected tropical diseases (NTDs). NTDs are a group of communicable diseases that are described as infections that have a high prevalence among people in poverty. Indeed, the disease burden of trachoma prevails in the tropical and sub-tropical regions of Africa [4]. These areas often consist of lower-income countries where there is poor access to clean water, overpopulated regions, and a history of inadequate funding of eradication programmes.

In 1998, the World Health Organisation (WHO) launched a new goal known as ‘Global Elimination of blinding Trachoma by 2020’ (GET2020). Alongside this goal, the WHO also devised a strategy to help tackle and control the disease; this involved surgery for trichiasis, antibiotic treatment, facial cleanliness, and environmental improvement (SAFE) plan to combat trachoma [5].

Establishing GET2020 has set the precedence for countries to take action against trachoma. Consequently, the incidence of trachoma has decreased globally with it being eradicated as a public health concern in some countries such as Cambodia and Ghana [1]. Nonetheless, the deadline for this objective has now arrived but trachoma remains a major concern in over 40 countries, including Nigeria.

Following the formation of GET2020, Nigeria quickly became a signatory of the programme and announced aims to improve the availability of water, sanitation, and health (WASH). Subsequently, they developed many ambitious initiatives such as the ‘open defecation free’ campaign to improve hygiene nationally [6]. Additionally, they have worked in close collaboration with non-governmental organisations (NGOs) and pharmaceutical companies to provide healthcare to those with, and at risk of, trachoma. However, despite great strides in reducing the incidence and prevalence of trachoma, Nigeria has been unable to achieve eradication by 2020 [7].

Trachoma remains a massive contributor to disability-adjusted life years lost in Nigeria. This review aims to evaluate many factors contributing to the inability of Nigeria to fulfil GET2020, as well as suggesting future steps required to eliminate trachoma.

## Review

Surgery

The main form of surgery for trichiasis is lid surgery. This is a relatively simple procedure which is often sight-saving when done timely and correctly [8]. Since the establishment of GET2020, great effort has been put into providing surgery at a community level. To overcome the limited availability of ophthalmologists, nurses have been trained to perform lid surgery in an effort to meet the clinical demand [9]. Research has shown no significant difference in clinical outcomes between nurses and doctors [10]. These surgical efforts have seen the prevalence of trichiasis decrease significantly across several states in Nigeria [7].

However, regardless of the availability of treatment, surgery is often poorly received. In Nigeria, poor surgery uptake is mainly due to financial constraints [11]. Furthermore, as surgery is largely aimed at preventing blindness, patients often do not perceive the need for treatment early on [12]. Moreover, sterilisation of equipment has been a big cause of concern among patients; particularly with regards to blood-borne diseases such as human immunodeficiency virus [13]. To counter this obstacle, the WHO has devised a surgical checklist which promotes the need to sterilise all equipment before any surgery [14].

Antibiotics

Antibiotics, especially azithromycin and tetracycline, are the main form of treatment for active trachoma [15]. Therefore, they are an essential component of any efforts to eliminate the disease. Historically, tetracycline ointment has been the treatment of choice for active trachoma. However, the discomfort associated with daily application of tetracycline to the eye resulted in reduced patient adherence [16]. In contrast, patients found oral azithromycin, taken once weekly, to be more convenient. Indeed, patient adherence rates and greater reductions in trachoma rates have been exhibited with the use of oral azithromycin as compared to tetracycline ointment. However, azithromycin is costly which makes widespread provision difficult. To address this, the pharmaceutical firm Pfizer Inc. co-founded the International Trachoma Initiative (ITI) in 1998 - a scheme that sees collaboration between ministries of health, local government associations, and NGOs [17]. Their goal is to provide large scale azithromycin, as well as aid in the development of the other facets of the SAFE strategy.

Infection with Chlamydia trachomatis is not immediately evident. Therefore, when only people with known active trachoma are treated, a reservoir of infection remains in the community [18]. Consequently, this increases the risk of reinfection. To combat this, the WHO recommends mass drug administration (MDA). MDA is the provision of drugs to whole populations in high-prevalence areas in order to treat active trachoma. MDA is to be administered in populations with a trachoma prevalence of greater than 10% among children aged between one and nine years, with a minimum of 80% of a community receiving antibiotics [19]. In 2010, 30 local government areas were surveyed for trachoma in Nigeria and, of the 30, seven were eligible to receive a donation from the ITI to provide MDA [20]. However, Nigeria has had difficulties in mapping out the districts most affected by trachoma. This is mainly due to a lack of funding and resources. It is highly likely there are other states that are eligible to receive MDA but have not yet been surveyed [13]. Nevertheless, Nigeria received its first donation from the ITI in 2010 to provide MDA for these seven local government areas.

Nigeria adopts an antibiotic delivery model whereby two to four community drug distributors (CDDs) are selected to volunteer and execute the MDA strategy. In order to assess the distribution of azithromycin within a community, CDDs must register each member of the community who receives treatment. However, the MDA target of 80% coverage is not always met. Certain states in Nigeria have been known to have an estimated antibiotic coverage of approximately 60% [20]. Furthermore, census data and population estimates are often inaccurate or unknown; leading to a discrepancy between the reported and true coverage rates achieved [21]. These difficulties have ultimately contributed to Nigeria’s shortcomings in achieving GET2020.

Considering these problems, different approaches must be undertaken to achieve timely elimination of trachoma. A proposed solution to mitigate inaccuracies in population coverage is to emulate the antibiotic delivery system used in Malawi, who achieved the GET2020 target in 2019 [22]. This approach involves registering the population prior to beginning an MDA within a village. Following registration, antibiotics are administered using a static site method whereby residents collect the antibiotics themselves. Next, the occupants are interviewed, allowing for accurate calculation of coverage rates. Following this, any people who did not receive initial treatment are offered antibiotics [23]. Emulating such proven strategies would likely lead to higher and more accurate coverage rates.

Water supply

Access to water encourages adequate hygiene and face-washing, and directly results in a reduced incidence of trachoma [24]. In Nigeria, there is a significant discrepancy in the availability of clean water between the north and south of the country which is largely due to the relatively poor rainfall in the north [25]. This is an important factor in explaining the comparatively higher trachoma rates seen in that region.

In rural Nigeria, hand pump boreholes are the main sources of water. However, it is estimated that only around half of these hand pumps are functional at a given time [26]. Therefore, even fewer people in rural areas have access to clean water than initially projected. Often, this is due to a lack of adequate maintenance.

Future projects focusing on both construction and maintenance of water amenities are being implemented to improve water supply. Currently, the Federal Ministry of Water Resources, who oversee WASH activities, are in the process of launching a new project known as Village Level Operation and Maintenance (VLOM) [27]. VLOM aims to construct hand pumps which can be predominantly maintained at a village level. Furthermore, the project aims to facilitate collaboration between the government and communities in relation to building and repairing boreholes. To make this possible, local mechanics and traders will be taught key repair skills in advance. Moreover, facility porters will be employed from each community with the responsibility of maintaining water and cleaning facilities. With these strategies in place to improve water supply, a rise in hygienic practices is likely to follow, resulting in a decrease in trachoma rates [24].

Environmental factors

Eye-seeking flies, such as Musca sorbens, are responsible for a substantial proportion of trachoma transmission. These insects tend to breed in human faeces and thus prevail in areas where open defecation is common practice [28]. In Nigeria, over 20% of the population practice open defecation, with higher rates observed in the north of the country where trachoma is also more prevalent [6]. A lack of latrines is the central factor for the high rates of open defecation in these communities. To combat the issue, Nigeria has launched an ‘Open Defecation Free’ campaign [26]. The end goal of this campaign is to provide access to sanitation, including lavatories with soap, to all Nigerians, especially in rural areas. Studies have shown that access to basic pit latrines lead to a significant reduction in the numbers of eye-seeking flies, correlating with an overall reduction in trachoma [29,30].

However, water and sanitation are heavily dependent on the behavioural norms and cultural beliefs of a community. Within Nigeria, many diseases are often assumed to be related to the spiritual world and superstitions [31]. Consequently, these beliefs have slowed the adoption of cleanliness measures in Nigeria as a means to combat water-related diseases. This has led to unhygienic routines, such as open defecation, to remain common practice even when pit latrines are available.

To tackle this, Nigeria has tried to implement community-led total sanitation (CLTS) approaches. The primary aim of CLTS is to educate a community and raise awareness about the consequences of poor hygiene [32]. CLTS has been proven to be very successful in other countries, such as India. India made significant progress towards being open defecation free following the launch of the ‘Total Sanitation Campaign’ [33]. This programme promoted and necessitated the need for individual latrines to be built within households. Furthermore, greater awareness was raised through media and community campaigns. Latrine use has increased by 30% in studied populations in India, largely as a result of these schemes [34]. The Nigerian government has stated that they will enter a ‘technical cooperation’ with the committee from India to adopt a similar approach [35]. If maintained, Nigeria may be able to replicate the results observed in India and further reduce rates of trachoma transmission.

Multi-pronged approach

There is an interdependent nature between the aforementioned approaches aimed at decreasing trachoma prevalence. Repeated single-armed strategies are ineffective in controlling and reducing trachoma rates [36]. Several studies have shown that antibiotic treatment alone is insufficient to eliminate trachoma and allows a reservoir of Chlamydia trachomatis to persist within the community. Indeed, families are significantly less likely to be re-infected by trachoma if they regularly participated in hygienic habits after antibiotic therapy [37]. Therefore, cross-sector collaboration bringing together the multiple facets of trachoma control is required to achieve eradication.

## Conclusions

Nigeria has taken massive strides towards reducing trachoma prevalence. However, in contrast to a number of other countries, the WHO’s GET2020 target has not been achieved. National trachoma treatment has been undertaken in Nigeria following years of neglect. However, it is clear that future interventions will have to be intensified to meet forthcoming targets. Current approaches are based on the SAFE strategy with a particular focus on improving water access and hygiene through WASH campaigns. Furthermore, great advances in drug availability have been undertaken through the ITI, which sees large scale antibiotics supplied by Pfizer Inc. Each strategy has aided in trachoma prevention in the country, but often it is a lack of cooperation between different sectors that lead to shortcomings in trachoma control. If trachoma eradication is to be achieved, further plans must see these sectors augment their strategies and work in cooperation.
